# Effects of miRNA-149-5p and Platelet-Activating Factor-Receptor Signaling on the Growth and Targeted Therapy Response on Lung Cancer Cells

**DOI:** 10.3390/ijms23126772

**Published:** 2022-06-17

**Authors:** Shreepa J. Chauhan, Anita Thyagarajan, Ravi P. Sahu

**Affiliations:** Department of Pharmacology and Toxicology, Boonshoft School of Medicine Wright State University, Dayton, OH 45345, USA; chauhan.22@wright.edu

**Keywords:** lung cancer, miR-149-5p, platelet-activating factor receptor, targeted therapy

## Abstract

Accumulating evidence indicates that microRNAs (miRs) play critical roles in essentially all biological processes and their altered expression has been documented in various disease conditions, including human malignancies. Although several cellular mechanisms have been identified in mediating the effects of miRs, the involvement of G-protein-coupled, platelet-activating factor-receptor (PAFR) signaling in miR-149-5p-induced effects on lung cancer growth and therapeutic potential has not been studied. To that end, we first evaluated the functional significance of PAFR and miR-149-5p in A549 and H1299 human non-small cell lung cancer (NSCLC) cell lines. We observed that these tumor lines express endogenous PAFR and miR-149-5p and that PAFR activation by PAF agonist (CPAF) significantly increased, whereas miR-149-5p mimic transfection inhibited cell proliferation in a dose-dependent manner. Interestingly, miR-149-5p mimic significantly attenuated CPAF-mediated increased proliferation of NSCLC cells, as confirmed by miR-149-5p, cyclin D1, and forkhead box protein M1 (FOXM1) expression analysis via qPCR. Our next studies examined PAFR- and miR-149-5p-mediated effects on targeted therapy (i.e., erlotinib and gefitinib) responses. We observed that erlotinib and gefitinib inhibited A549 and H1299 cell survival in a dose- and time-dependent manner, and CPAF significantly blocked this effect. These findings indicate that miR-149-5p blocks PAFR-mediated increased cell proliferation, and PAFR activation attenuates the cytotoxic effects of targeted therapy.

## 1. Introduction

MicroRNAs (miRs) are conserved single-stranded small (i.e., 19–22 nucleotides) noncoding RNAs, which have been shown to play important roles in essentially all biological and pathophysiological processes, including cancer [1,2]. Importantly, miRs are found to be aberrantly expressed in several human cancers, including lung cancer, and their upregulation and downregulation have been documented to either promote or suppress cancer growth [3,4]. Notably, cancer-specific miR expression signatures have been linked with several clinic-pathological variables, including tumor stages/metastasis, disease reoccurrence, treatment resistance, and patient survival [3]. Several miRs can be expressed endogenously in any given tumor line, and their expression levels (e.g., up- or downregulation) can help to characterize its mechanisms involved in supporting cancer growth and/or impeding the efficacy of cancer therapies [5,6,7,8,9,10].

In lung cancer models, unlike most of the miRs that have been reported for their oncogenic effects, miR-149 has been shown to function as a tumor suppressor via its ability to induce apoptosis [11,12,13]. Notably, non-small-cell lung carcinoma (NSCLC) accounts for the majority of the lung cancer cases diagnosed; however, the mechanisms affecting cancer growth and the cytotoxic responses of currently explored targeted therapy such as receptor tyrosine kinase inhibitors (TKIs; erlotinib and gefitinib) are yet to be fully explored [14,15,16,17]. In particular, studies, including ours, have demonstrated the critical involvement of a widely expressed G-protein-coupled, platelet-activating factor receptor (PAFR) pathway in augmenting tumor growth, including in lung cancer, and impeding the efficacy of cancer therapies [18,19,20,21]. However, the role of miR-149-5p in PAFR-mediated effects on lung cancer growth or therapeutic potential has not been studied.

In the current study, we utilized PAFR-expressing cellular NSCLC models, pharmacological agents, and molecular biology approaches to determine the effects of miR-149 and PAFR on the growth, as well as the cytotoxic response, of erlotinib and gefitinib. Our studies demonstrated that PAFR activation augmented, whereas miR-149-5p decreased, the proliferation of NSCLC cells in a dose-dependent manner. Interestingly, miR-149-5p attenuated PAFR activation-mediated increased cell proliferation. Erlotinib and gefitinib inhibited the survival of A549 and H1299 cell lines in a dose-dependent manner, and PAFR activation attenuated the cytotoxic response of these targeted therapies.

## 2. Results

### 2.1. Functional PAFR Activation Augments the Proliferation of NSCLC Cell Lines

**Figure 1:**
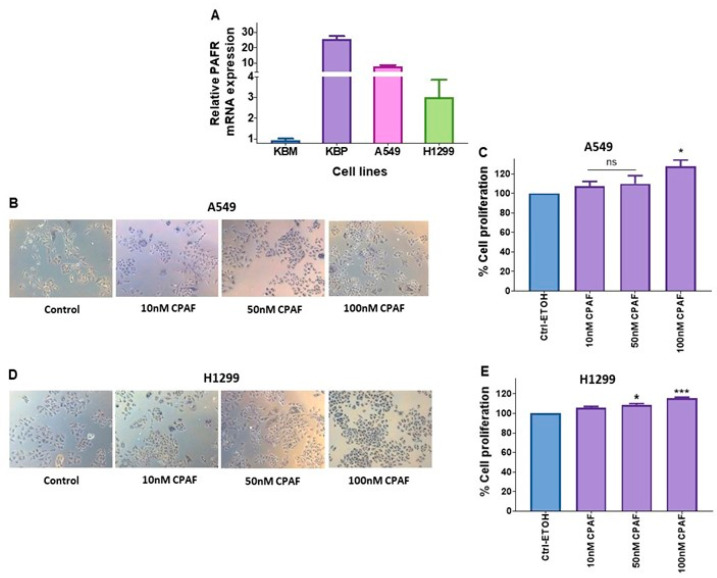
Evaluation of functional PAFR expression in human NSCLC cell lines. (**A**) The PAFR mRNA expression analysis in A549 and H1299 cell lines was undertaken via qPCR using KBP and KBM cells as positive and negative controls. (**B**–**E**) A549 and H1299 cell lines were treated with vehicle (0.1% ETOH) as control (Ctrl) or CPAF at given doses, and cultured for 48 h. The cell proliferation was analyzed via MTT (**B**,**D**); the representative pictures are shown) and CV (**C**,**E**) assays. Data are expressed as the mean ± SE of three separate experiments. A statistically significant difference (* = *p* < 0.05; *** = *p* < 0.001) was observed by means of Student’s *t*-test between the control and 100 nM CPAF-treated groups.

Our first studies began with re-evaluating the PAFR expression in A549 and H1299 NSCLC cell lines via qPCR. As shown in Figure 1A, both these cell lines express PAFR as compared to the stably PAFR-expressing KBP and PAFR-deficient KBM cell lines, used as positive and negative controls. To determine the functional significance of the PAFR expression, we then tested the effect of a known PAFR agonist, carbamyl-PAF (CPAF). We observed that CPAF treatment significantly enhanced the in vitro proliferation of both A549 and H1299 cell lines in a dose-dependent manner as compared to vehicle controls, as analyzed via MTT or crystal violet (CV) assays (Figure 1B–E). The timeline of the experimental analysis is described in the Materials and Methods section for each assay, in respective figure legends, and is also illustrated in Supplementary Figure S1. Given that similar cell-proliferative effects of CPAF were noted in A549 and H1299 cell lines with a significant change at a dose of 100 nM CPAF, we used A549 cells for our subsequent studies, as this is a widely-used cellular model of NSCLC. Since the data obtained through both the MTT and CV assays were similar, we used the CV assay to measure cell proliferation in the subsequent experiments.

### 2.2. miR-149-5p Inhibits the Proliferation of A549 Cells in a Dose-Dependent Manner

**Figure 2:**
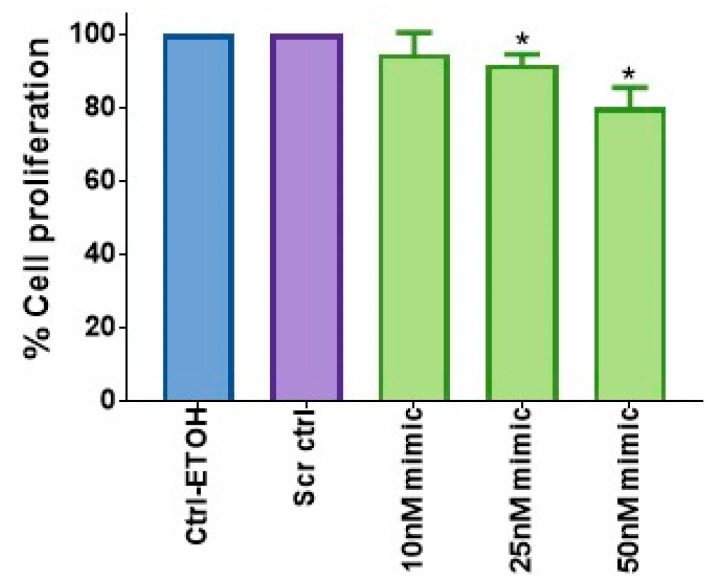
The effect of miR-149-5p overexpression on the proliferation of the A549 cell line. A549 cells were treated with vehicle (0.1% ETOH) as a control, or transfected with Scr ctrl as a transfection control, or with different doses of miR-149-5p mimics. After 48 h of incubation, the cell proliferation was analyzed via the CV assay. Data are expressed as the mean ± SE of three separate experiments. Statistically significant differences (* = *p* < 0.05) were observed, according to Student’s *t*-test, between the control groups and cells treated with various doses of the miR-149-5p mimic.

We then tested the effect of miR-149-5p on the growth of A549 cells. We demonstrated that transfection of miR-149-5p specific mimics reduced the in vitro proliferation of A549 cells in a dose-dependent manner compared to the vehicle control or scrambled-miRNA control (Scr ctrl) transfected cells (Figure 2). These findings verified the growth inhibitory (tumor suppressive) activity of miR-149-5p.

### 2.3. miR-149-5p Overexpression Attenuates CPAF-Mediated Increased Proliferation of A549 Cells

**Figure 3:**
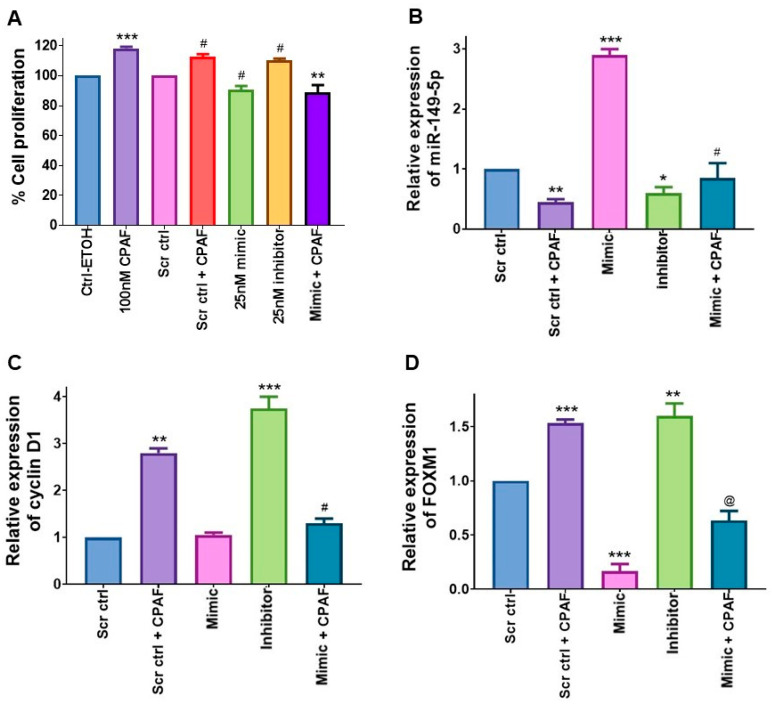
The effect of miR-149-5p overexpression on CPAF-mediated increased A549 cell proliferation. (**A**) A549 cells were treated with vehicle (0.1% ETOH) and 100 nM CPAF as negative and positive controls, or transfected with a 25 nM dose of Scr ctrl (± CPAF) for transfection control, or a 25 nM dose of miR-149-5p mimic (± CPAF), and an miR-149-5p-specific inhibitor. After 48 h of incubation, the cell proliferation was analyzed via the CV assay. Data are expressed as the mean ± SE of three separate experiments. Statistically significant differences were observed, according to Student’s *t*-test, between Ctrl-ETOH vs. CPAF (*** = *p* < 0.001), Scr ctrl vs. Scr ctrl + CPAF (# = *p* < 0.01), Scr ctrl vs. mimic (# = *p* < 0.05), Scr ctrl vs. inhibitor (# = *p* < 0.01), and CPAF vs. mimic + CPAF (** = *p* < 0.01). (**B**) Analysis of miR-149-5p expression was carried out via qPCR, using CPAF, miR-149-5p mimic, miR-149-5p inhibitor, and miR-149-5p mimic with CPAF. Data are expressed as the mean ± SE of three independent experiments. Statistically significant differences were observed, based on Student’s *t*-test, between Scr ctrl vs. Scr ctrl + CPAF (** = *p* < 0.01) and Scr ctrl vs. inhibitor (* = *p* < 0.05), and based on one-way ANOVA with the Bonferroni post hoc test between Scr ctrl vs. mimic (*** = *p* < 0.001) and mimic vs. mimic + CPAF (# = *p* < 0.001). (**C**) Analysis of Cyclin D1 was performed via qPCR with CPAF miR-149-5p mimic, miR-149-5p inhibitor, and mimic with CPAF. Data are expressed as the mean ± SE of three separate experiments. Statistically significant differences were observed, according to one-way ANOVA with the Bonferroni post hoc test, between Scr ctrl vs. Scr ctrl + CPAF (** = *p* < 0.01), Scr ctrl vs. inhibitor (*** = *p* < 0.001) and via Student’s *t*-test between Scr ctrl + CPAF vs. mimic + CPAF (# = *p* < 0.01). (**D**) FOXM1 expression analysis was carried out via qPCR, using CPAF, miR-149-5p mimic, miR-149-5p inhibitor, and mimic with CPAF. Data are expressed as the mean ± SE of three independent experiments. Statistically significant differences were observed between Scr ctrl vs. Scr ctrl + CPAF (*** = *p* < 0.001), Scr ctrl vs. mimic (*** = *p* < 0.001), Scr ctrl vs. inhibitor (** = *p* < 0.01), and Scr ctrl + CPAF vs. mimic + CPAF (@ = *p* < 0.001).

We next tested whether miR-149-5p overexpression can overcome the cell proliferating effects of CPAF or vice versa. To that end, A549 cells were transfected with Scr ctrl or miR-149-5p mimic, and after 6 h of incubation, treated with or without CPAF, and cell proliferation was measured via the CV assay. Similar to our previous findings, CPAF treatment increased, and miR-149-5p mimic reduced, the proliferation of A549 cells, which verified the reproducibility of the data (Figure 3A). In addition, transfection with the miR-149-5p-specific inhibitor rescued miR-149-5p mimic-induced effects, confirming the specificity of miR-149-5p in the decreased growth of A549 cells (Figure 3A). Interestingly, miR-149-5p overexpression significantly blocked the CPAF-mediated increased proliferation of A549 cells (Figure 3A). Importantly, qPCR analysis of miR-149-5p in similar experimental settings demonstrated that CPAF treatment reduces, and mimic increases, miR-149-5p expression, and that the combination of mimic + CPAF exhibits decreased miR-149-5p expression (Figure 3B). The data indicate that while PAFR activation can reduce the endogenous expression of miR-149-5p, its overexpression via the mimic was able to overcome CPAF-mediated decreased expression of miR-149-5p (Figure 3B). In the same context, these findings are also supported by the results shown in Figure 3A, as described above.

We also analyzed the cell cycle proliferative marker cyclin D1 as a target of PAFR- and miR-149-5p-induced effects via the qPCR assay. Our studies demonstrated that CPAF and the miR-149-5p inhibitor increase, and the miR-149-5p mimic reduces, cyclin D1 expression, and the combination of mimic + CPAF exhibits lower miR-149-5p expression (Figure 3C). These findings suggest that miR-149-5p can override the cell growth enhancing effect mediated by PAFR activation. Moreover, the expression of forkhead box protein M1 (FOXM1), a transcription factor with oncogenic activity and a known target of miR-149, was evaluated in this context. We observed that CPAF treatment significantly upregulated FOXM1 expression (Figure 3D), indicating a crosstalk of the PAFR signaling with FOXM1. The overexpression of miR-149-5p significantly downregulated, and its inhibition via miR-149-5p specific inhibitor rescued, FOXM1 expression (Figure 3D). These findings are consistent with the published report demonstrating that miR-149 overexpression inhibited FOXM1 expression, indicating that FOXM1 is a known target of miR-149 [12]. Importantly, CPAF-mediated increased expression of FOXM1 was significantly attenuated by the mimic (Figure 3D), indicating that miR-149-5p overexpression can override this PAFR activation-induced effect.

### 2.4. Effects of PAFR Antagonist on miR-149-5p-Mediated Reduced Cell Proliferation

**Figure 4:**
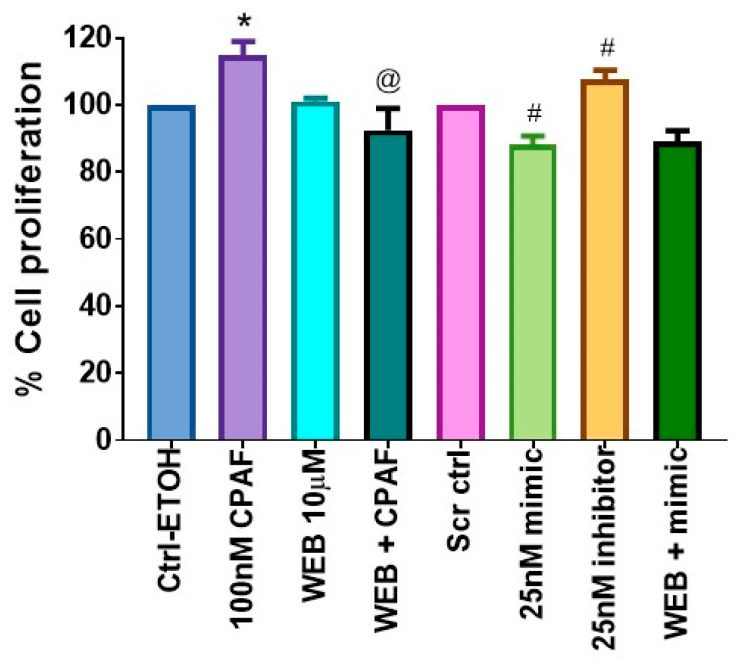
The effects of PAFR antagonist and miR-149-5p overexpression on A549 cell proliferation. A549 cells were pretreated with a PAFR antagonist, WEB2086 (10 µM, 1 h), followed by treatments with ± 0.1% ETOH as a vehicle and 100 nM CPAF as negative and positive controls, or transfected with a 25 nM dose of Scr ctrl (± CPAF) as a transfection control, or a 25 nM dose of miR-149-5p mimic (± CPAF) and miR-149-5p inhibitor. After 48 h of incubation, the cell proliferation was analyzed via the CV assay. Data are expressed as the mean ± SE of three separate experiments. Statistically significant differences were observed, according to Student’s *t*-test, between Ctrl-ETOH vs. CPAF (* = *p* < 0.05), CPAF vs. WEB + CPAF (@ = *p* < 0.05), Scr ctrl vs. mimic (# = *p* < 0.01), and Scr ctrl vs. inhibitor (# = *p* < 0.05).

To determine whether PAFR blockade can synergize with the miR-149-5p-mediated decreased cell proliferative effect, we tested the effects of a specific PAFR antagonist, the WEB2086 compound. For this purpose, A549 cells were pretreated with WEB2086 (10 µM) for 1 h, followed by treatment with or without CPAF (100 nM), or transfected with a 25 nM dose of Scr ctrl, miR-149-5p mimic, or miR-149-5p-specific inhibitor, and cultured for 48 h. Our studies demonstrated that although WEB2086 alone did not exert any effect, it significantly attenuated CPAF-mediated increased cell proliferation (Figure 4). Although significant differences were noted between control and CPAF, CPAF and WEB + CPAF, and scrambled control and miR-149-5p mimic or miR-149-5p inhibitor, there was no difference between miR-149-5p mimic and WEB + miR-149-5p mimic (Figure 4). These findings indicate that WEB2086 did not synergize miR-149-5p mediated effects in further inhibiting the proliferation of A549 cells.

### 2.5. Erlotinib and Gefitinib Inhibit the Growth of NSCLC Cell Lines in a Dose- and Time-Dependent Manner

**Figure 5:**
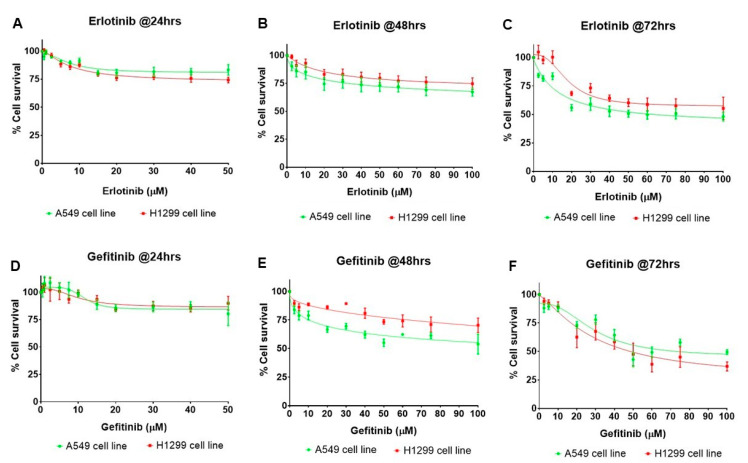
Effects of erlotinib and gefitinib on the survival of human NSCLC cells. (**A**–**F**) A549 and H1299 cells were treated with vehicle (0.1% DMSO) or various concentrations of erlotinib or gefitinib, and incubated for 24, 48, and 72 h. The cell survival was measured via the sulforhodamine B (SRB) assay. Data are expressed as the mean ± SE of three independent experiments and are presented as the percentage (%) of cell survival against erlotinib or gefitinib treatments.

To determine the effects of PAFR activation and miR-149-5p overexpression on the efficacy of targeted therapies in NSCLC cell lines, we first optimized the dose- and time-dependent cytotoxic responses of erlotinib and gefitinib using A549 and H1299 cell lines. These cell lines were treated with various reported doses of erlotinib and gefitinib [15,22,23,24,25,26] and incubated for 24, 48, and 72 h. We observed that both these drugs inhibited the survival of A549 and H1299 cell lines in a dose- and time-dependent manner, with the maximum effects observed at 72 h (Figure 5). Since there were no substantial differences between erlotinib and gefitinib-induced cytotoxicity in A549 and H1299 cell lines, we used the A549 cell line for our next experiments with these targeted therapies.

### 2.6. Effects of PAFR Activation, PAFR Antagonist, and miR-149-5p Overexpression on the Cytotoxic Responses of Erlotinib and Gefitinib

**Figure 6:**
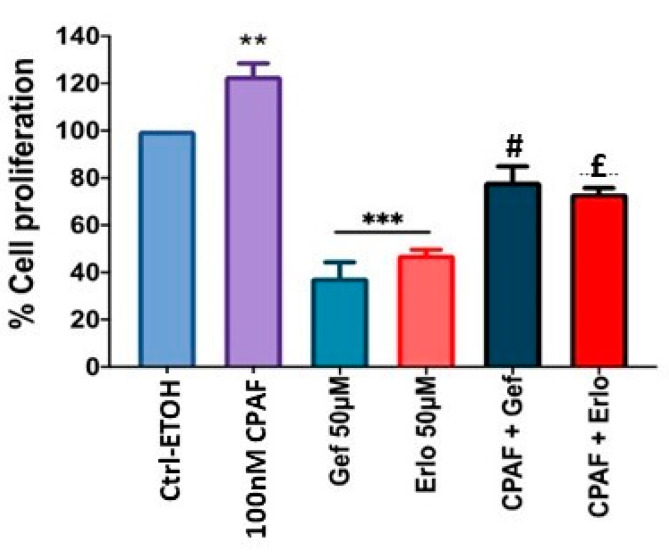
Effect of CPAF on targeted therapy-mediated decreased A549 cell proliferation. A549 cells were pre-treated with CPAF (100 nm, 1 h), followed by treatments with ± 0.1% ETOH, Gef or Erlo at given doses. After 72 h of incubation, the cell proliferation was analyzed via the CV assay. Data are expressed as the mean ± SE of three separate experiments. Statistically significant differences were observed, according to one-way ANOVA with the Bonferroni post hoc test, between Ctrl-ETOH and CPAF (** = *p* < 0.01), Ctrl-ETOH and Gef 50 µM or Erlo 50 µM (*** = *p* < 0.001), and according to Student’s *t*-test between Gef vs. CPAF + Gef (# = *p* < 0.01) or Erlo and CPAF + Erlo (£ = *p* < 0.001).

**Figure 7:**
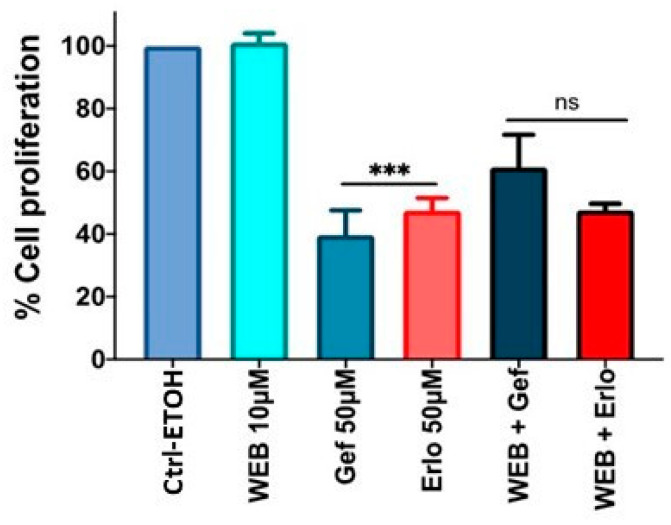
Effect of PAFR antagonist on targeted therapy-mediated decreased cell proliferative response. A549 cells were pretreated with a PAFR antagonist, WEB2086 (10 µM, 1 h), and then with ±Gef or Erlo (50 µM) and vehicle (0.1% ETOH). After 72 h of incubation, the cell proliferation was measured via the CV assay. Data are expressed as the mean ± SE of five separate experiments. Statistically significant differences were observed, via one-way ANOVA with the Bonferroni post hoc test, between Ctrl-ETOH and Gef 50 µM or Erlo 50 µM (*** = *p* < 0.001). “ns” denotes a non-significant change between Gef and WEB + Gef or Erlo and WEB + Erlo.

**Figure 8:**
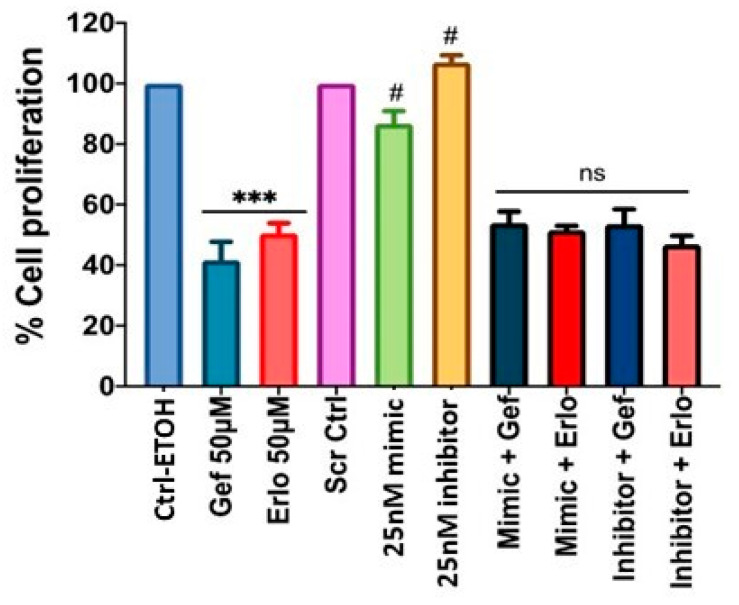
Effect of miR-149-5p mimic and miR-149-5p inhibitor on targeted therapy-induced decreased cell proliferation. A549 cells were pre-transfected with miR-149-5p mimic or miR-149-5p inhibitor (25 nm, 6 h), followed by treatments with ±Gef or Erlo (50 µM) and vehicle (0.1% ETOH) or scrambled control (25 nM). After 72 h of incubation, the cell proliferation was analyzed via the CV assay. Data are expressed as the mean ± SE of four separate experiments. Statistically significant differences were observed, via one-way ANOVA with the Bonferroni post hoc test, between Ctrl-ETOH and Gef or Erlo (*** = *p* < 0.001), and, via Student’s *t*-test, between Scr ctrl vs. mimic (# = *p* < 0.05) and Scr ctrl vs. inhibitor (# = *p* < 0.05). “ns” denotes a non-significant change between mimic + Gef, or mimic + Erlo with Gef or Erlo alone treatments, and a similar response was observed with inhibitor + Gef or inhibitor + Erlo.

**Figure 9:**
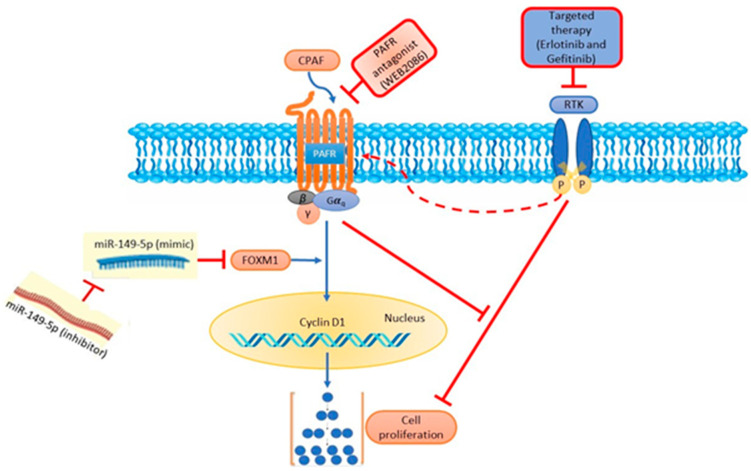
Schematic representation of PAFR- and miR-149-5p-mediated effects on the growth of NSCLC cells and/or cytotoxic responses of targeted therapies. In this model, the PAFR activation (via CPAF) induces an increased proliferation of NSCLC cells by an upregulation of cyclin D1 and FOXM1, in a process which is blocked by the overexpression of miR-149-5p and PAFR antagonist. Importantly, miR-149-5p mimic-mediated effects are blocked by the miR-149-5p-specific inhibitor, comparable to the PAFR activation-induced effects, indicating their crosstalk and antagonizing functions. Targeted therapies decrease the proliferation of NSCLC cells in a process blocked by PAFR activation.

Using the optimum (i.e., 50 µM at 72 h) dose of gefitinib and erlotinib, we determined the effects of PAFR activation, PAFR antagonists, and miR-149-5p overexpression. For this purpose, A549 cells were pretreated with CPAF (100 nM) or WEB2086 (10 µM) for 1 h, or transfected with Scr ctrl or miR-149-5p mimic, and the miR-149-5p inhibitor. Following 6 h of incubation, the cells were treated with or without CPAF, gefitinib, and erlotinib (50 µM), and were cultured for 72 h. Our studies demonstrated that PAFR activation significantly reversed gefitinib and erlotinib-mediated decreased cell proliferation (Figure 6). However, neither the PAFR antagonist (Figure 7) nor the miR-149-5p mimic or the miR-149-5p inhibitor (Figure 8) exerted any significant effects on the cytotoxic response of gefitinib and erlotinib as compared to the treatments with these agents alone. The schematic representation of PAFR- and miR-149-5p-mediated effects on the growth of NSCLC cells and/or cytotoxic responses of targeted therapies is shown as Figure 9.

## 3. Discussion

Several human malignancies, including lung cancer, express functional PAFR, and high tumoral PAFR expression has been positively correlated with increased tumor invasiveness/stages and decreased survival of esophageal, breast, and lung cancer patients [27,28]. Studies, including ours, have demonstrated that PAFR activation enhances the in vitro proliferation and in vivo tumor growth of NSCLC cell lines via mechanisms involving the signal transducer and activator of transcription 3 (STAT3) signaling [18,29]. In addition, host PAFR activation has been shown to impede the efficacy of cancer therapies in various experimental tumor models [19,20,21]. However, the question of whether miRs can mediate these PAFR-induced effects has not been studied.

Accumulating evidence suggests that depending upon cancer types, similar miRs can function either as tumor suppressors or oncogenes [2,3,4]. To this end, numerous studies have demonstrated that miRs play crucial roles in neocarcinogenesis and the progression of human cancers, including NSCLC [15,30]. Importantly, miRs, including miR-149, have been shown to be endogenously expressed in tumor cell lines, and their expression levels (e.g., up- or downregulation) can help to characterize their mechanisms in supporting cancer growth and/or impacting the efficacy of cancer therapies [11,12,13]. Thus, miRs are considered potential targets or biomarkers in cancer therapeutics. Significantly, miR-149 has been shown to function as an oncogene in hepatocellular carcinoma, breast cancer, renal cell carcinoma, gastric cancer, pancreatic cancer, and neuroblastoma via its ability to crosstalk with and upregulate various oncogenic signaling cascades [11,12]. However, in lung cancer models, the tumor suppressive effects of miR-149 have been documented [11,12,13]. Low expression of miR-149 has been documented in NSCLC tissue and is positively correlated with tumor size and metastasis [11]. Importantly, reduced miR-149 expression in tumor tissue was found to be associated with lower overall survival and disease-free survival rates compared to the high miR-149 expression group in NSCLC patients [11].

Based upon this rationale, in the current study we sought to determine the effects of miR-149-5p and PAFR on the growth and cytotoxic responses of targeted therapies using NSCLC cellular models [31]. We hypothesized that the tumor suppressive activity of miR-149-5p would circumvent the PAFR-mediated growth enhancing effect or reduce the therapeutic response in our lung cancer model. We began our study by re-evaluating PAFR and miR-149 expression, as well as their functional significance. As previously reported, we found that both A549 and H1299 cell lines express endogenous PAFR and miR-149 [11,29]. The functional studies confirmed that PAFR activation induced an increased proliferation of both the cell lines in a dose-dependent manner, similarly to what has been shown in various tumor models [19,29,31]. Consistent with the previous findings, in our study we demonstrated that transfection with miR-149-specific mimics reduced the proliferation of A549 cells in a dose-dependent manner compared to the normal control or scrambled-miRNA transfection control, verifying its tumor suppressor activity.

We then determined whether miR-149-5p can override the cell proliferative effect of PAFR activation, demonstrating that the miR-149-5p mimic significantly blocked CPAF-mediated increased proliferation of A549 cells. Furthermore, qPCR analysis of miR-149-5p showed that CPAF treatment reduces, and the mimic increases, miR-149-5p expression, and that the mimic also significantly reversed CPAF-induced decreased miR-149-5p expression. These findings are supported by the analysis of the cell cycle proliferative marker (cyclin D1), which served as a target for PAFR and miR-149 [11,12]. The results indicated that treatments with CPAF and the miR-149-5p-specific inhibitor significantly increased, whereas miR-149-5p mimic reduced, cyclin D1 expression. Interestingly, the miR-149-5p mimic significantly attenuated CPAF-mediated increased cyclin D1 expression.

Given that miR-149 interacts with cyclin D1 and has also been shown to directly target FOXM1, we next determined the effects of PAFR activation and miR-149-5p overexpression on the levels of FOXM1 expression to support our findings. Notably, FOXM1 is an oncogenic transcription factor of the forkhead family that plays critical roles in regulating cell cycle progression leading to mitosis [32,33]. Notably, the deregulation (i.e., aberrant upregulation) of FOXM1 has been reported to be a key driver of neo-oncogenesis and cancer progression, and was found to be associated with poor prognosis in several human malignancies, including lung cancer [34,35]. The data demonstrated that CPAF treatment significantly increased FOXM1 expression, indicating its crosstalk with PAFR. Treatment with the mimic significantly inhibited FOXM1 expression, which is consistent with the published report that miR-149 targets FOXM1 [12]. Importantly, overexpression of miR-149-5p significantly attenuated CPAF-induced increased FOXM1 expression. These findings suggest that miR-149-5p can override the growth enhancing effects of NSCLC cells mediated by the PAFR activation.

We then determined the effects of the PAFR antagonist and miR-149-5p overexpression on the growth of NSCLC cells. We observed that the PAFR antagonist, which blocked CPAF-mediated increased cell proliferation, did not exert synergistic effect in reducing cell proliferation in combination with the miR-149-5p mimic. One possible explanation for this is that the effect of the PAFR antagonist could be dependent on the dose, as therapeutic effects need higher doses than those required to antagonize the receptor. This remains one of the limitations of this study, as higher doses of WEB2086 with the miR-149-5p mimic could result in exaggerated cell death, and thus could induce an off-target effect. In our future studies we will explore testing the efficacy of other PAFR antagonists with the miR-149-5p mimic in NSCLC models.

Therapeutic approaches to lung cancer depend on the cancer stage, and include surgical resection, chemotherapy, radiation therapy, and targeted therapies. However, among other targeted therapies, tyrosine kinase inhibitors such as erlotinib or gefitinib are currently being used to treat NSCLC patients who have mutated or overactivated EGFR signaling in tumor cells, which play an important role in sustained cell proliferation [22,24,25,26]. To that end, we tested the effects of erlotinib or gefitinib and found that both these agents inhibited the survival of A549 and H1299 cell lines in a dose- and time-dependent manner. Notably, PAFR activation was able to significantly block targeted-therapy-mediated decreased cell proliferation. However, the PAFR antagonist, miR-149-5p mimic, and miR-149-5p inhibitor did not exert any significant effects on the cytotoxic responses of these therapeutic agents. The reason for this discrepancy is not clear at this time, which remains another limitation of our project and requires future exploration. In summary, in the present study we evaluated the potential roles of miR-149-5p and PAFR signaling in relation to lung cancer growth and the cytotoxic response of targeted therapy, and found that miR-149 overcame PAFR activation-induced increased cell proliferation, and PAFR activation attenuated the cytotoxic response of targeted therapy. These findings indicate that PAFR signaling has a potential role in modulating the cytotoxic response of targeted therapy.

In conclusion, the results of this study demonstrated that miR-149-5p overexpression blocks the proliferation of NSCLC cells induced by the PAFR activation. These findings were confirmed through the analyses of cyclin D1 and FOXM1 expression, the markers of cell cycle progression. Although PAFR and miR-149-5p have been shown to interact with cyclin D1, and FOXM1 is the direct target of miR-149-5p, the crosstalk between PAFR signaling and miR-149-5p and FOXM1 has not been studied before. Our study results demonstrate that PAFR activation reduces the endogenous expression of miR-149-5p, and induces cyclin D1 and FOXM1 expression. Importantly, miR-149-5p overexpression restores the decreased expression of the endogenous miR-149-5p level and attenuates the increased expression of cyclin D1 and FOXM1 induced by the PAFR activation. Interestingly, PAFR activation was found to reduce the cytotoxic effects of targeted therapy; however, the PAFR antagonist and miR-149-5p overexpression exerted no effects on targeted therapy-mediated decreased proliferation of NSCLC cells. Overall, this study highlights the importance of PAFR signaling in miR-149-5p-mediated effects in NSCLC cells and thus demonstrates the need for further exploration of this axis in relation to the therapeutic potential of targeted therapy in experimental lung cancer models.

## 4. Materials and Methods

### 4.1. Reagents and Cells

The culture media were purchased from GE Healthcare Biosciences (Marlborough, MA, USA). The PAFR agonist, carbamyl-PAF (CPAF), PAFR antagonist (WEB2086), erlotinib, and gefitinib were obtained from Cayman Chemicals Co. (Ann Arbor, MI, USA). The RNA extraction kit was purchased from Invitrogen Life Technologies (Carlsbad, CA, USA). The miR-149, High-Capacity cDNA Reverse Transcription kits, SYBR green, and PCR reagents were purchased from Applied Biosystems (Carlsbad, CA, USA). The PAFR, miR-149-5p, cyclin D1, β-actin, 18s, U6, and GAPDH primers were also procured from Applied Biosystems (Carlsbad, CA, USA). The primers for the FOXM1 gene were selected from the published reports [36,37]. The MTT assay kit was purchased from Millipore Sigma (St. Louis, MO, USA). PAFR-expressing (KBP) and PAFR-deficient (KBM) human cell lines were used as positive and negative controls to evaluate PAFR expression in A549 and H1299 cell lines, and these were cultured as per our previous reports [20,21] under our WSU IBC protocol number #369. KBP cells were created from the PAFR-negative human epidermal KB cell line via transduction with the MSCV2.1 retroviral vector, containing human PAFR cDNA, whereas KBM cells were transduced with MSCV2.1 retroviral vector [38]. The human lung epithelial carcinoma A549 and H1299 NSCLC cell lines were grown as previously reported [31].

### 4.2. Quantitative Real-Time PCR (qPCR) Analysis

For the PAFR mRNA expression analysis, A549, H1299, KBM, and KBP cell lines were homogenized using an RLT buffer containing β-mercaptoethanol (Sigma-Aldrich, St. Louis, MO, USA) in a bullet blender (Next Advance, Inc., Averill Park, NY, USA) and carbide beads. Total RNA was extracted from these cell lines using an RNaesy Plus mini kit as per the manual’s protocol, and quantified with Nanodrop 2000c (Thermo Scientific, Vernon Hills, IL, USA). For the analysis of miR-149-5p, cyclin D1, and FOXM1 mRNA expression, A549 cells were transfected with or without the miR-149-5p mimic or inhibitor and incubated for 6 h, followed by treatment with or without CPAF and incubated for 48 h. The control cells were transfected with Scr ctrl. Total RNA samples were extracted as described above. A High-Capacity cDNA Reverse Transcription kit containing random hexamers was used to transcribe RNA samples to cDNA as per the manufacturer’s instructions, and a SYBR green-based quantitative qPCR method was performed as per the manufacturer’s instructions and as previously reported [31,39]. The fluorescence was detected using a StepOne Real-Time PCR machine (Applied Biosystems, Foster City, CA, USA). We used GAPDH, U6, and 18s as endogenous controls to normalize PAFR, miR-149, and cyclin D1 expression. The quantification of the PCR product was analyzed using the 2^−∆∆Ct^ method.

### 4.3. MTT Assay

Cell proliferation was examined using the MTT assay kit, using the standard protocol from the user’s manual. To evaluate the effect of functional PAFR expression, 8 × 10^3^ cells were seeded in 48 well plates and treated with or without CPAF and incubated for 48 h; then cell proliferation was analyzed. To determine the effect of miR-149-5p, cells were transfected with miR-149-5p-specific mimics and inhibitors using Lipofectamine 3000 transfection kit (Invitrogen, Carlsbad, CA, USA) and the protocol from the user manual. After 6 h of transfection, cells were treated with ethanol as a vehicle control and 100 nM CPAF and incubated for 48 h. Then, cells were stained with MTT, and following 3 h of incubation, the MTT stain was solubilized with 10% SDS in 0.01 M HCl, and absorbance values were taken at 570 nm with SYNERGY H1 microplate reader (BioTek Instruments, Inc., Winooski, VT, USA). Finally, the values of treated samples were normalized with the control samples to determine the relative percentage of cell proliferation.

### 4.4. Crystal Violet Assay

NSCLC cell proliferation was also examined via the crystal violet (CV; Sigma-Aldrich, St. Louis, MO, USA) assay [40]. In this assay, following the transfection, treatments, and incubation as per the MTT assay, cells were washed twice with sterile PBS and fixed with 100% ice-cold methanol, and the plates were incubated at −20 °C for 30 min. The methanol was decanted and cells were stained with 0.4% CV solution and incubated at room temperature for 5–10 min. The plates were rinsed using distilled water and air-dried. Then, the CV stain was solubilized with 1% SDS solution, and the absorbance was measured at 540 nm with SYNERGY H1 microplate reader. Finally, the relative percentage of cell proliferation was determined as described above.

### 4.5. Cell Survival Assay

Cell survival was measured via the sulforhodamine B (SRB; Sigma-Aldrich, St. Louis, MO, USA) assay as previously reported [39]. Briefly, cells were seeded in 96-well plates at 5 × 10^3^ cell density per well and treated with 0.1% DMSO as control or various concentrations of erlotinib and gefitinib (1–100 mM). The cell survival was assessed at 24, 48, and 72 h. Finally, the absorbance was read at 570 nm with SYNERGY H1 microplate reader, and the treated groups were normalized with the control group.

### 4.6. Statistical Analysis

Statistical analysis was assessed using GraphPad Prism software version 7.0 (GraphPad software, San Diego, CA, USA). All in vitro experiments were performed in triplicate, and repeated independently at least three times. Data were analyzed by Student’s *t*-test or one-way ANOVA with the post hoc Bonferroni multiple comparison tests. *p*-values of less than 0.05 were considered to indicate a statistically significant difference between two groups.

## Data Availability

The data supporting the findings of this study will be available on request.

## Supplementary Materials

The following supporting information can be downloaded at: https://www.mdpi.com/article/10.3390/ijms23126772/s1.

## Abbreviations

PAFR	platelet-activating factor receptor
miR	microRNA
Scr ctrl	scrambled-miRNA control
NSCLC	non-small-cell lung cancer
CPAF	carbamyl-PAF
TKIs	tyrosine kinase inhibitors
Gef	gefitinib
Erlo	erlotinib
CV	crystal violet
DMSO	dimethyl sulfoxide
ETOH	ethanol
SRB	sulforhodamine B
FOXM1	forkhead box protein M1
STAT3	signal transducer and activator of transcription 3

## References

[1] Catalanotto C., Cogoni C., Zardo G. (2016). MicroRNA in Control of Gene Expression: An Overview of Nuclear Functions. Int. J. Mol. Sci..

[2] Adams B.D., Kasinski A.L., Slack F.J. (2014). Aberrant regulation and function of microRNAs in cancer. Curr. Biol..

[3] Li M., Li J., Ding X., He M., Cheng S.Y. (2010). microRNA and cancer. AAPS J..

[4] Wu K.L., Tsai Y.M., Lien C.T., Kuo P.L., Hung A.J. (2019). The Roles of MicroRNA in Lung Cancer. Int. J. Mol. Sci..

[5] Nie H., Xie X., Zhang D., Zhou Y., Li B., Li F., Li F., Cheng Y., Mei H., Meng H. (2020). Use of lung-specific exosomes for miRNA-126 delivery in non-small cell lung cancer. Nanoscale.

[6] Chen J., Liu A., Wang Z., Wang B., Chai X., Lu W., Cao T., Li R., Wu M., Lu Z. (2020). LINC00173.v1 promotes angiogenesis and progression of lung squamous cell carcinoma by sponging miR-511-5p to regulate VEGFA expression. Mol. Cancer.

[7] Padda J., Khalid K., Khedr A., Patel V., Al-Ewaidat O.A., Tasnim F., Padda S., Cooper A.C., Jean-Charles G. (2021). Exosome-Derived microRNA: Efficacy in Cancer. Cureus.

[8] Ali Syeda Z., Langden S., Munkhzul C., Lee M., Song S.J. (2020). Regulatory Mechanism of MicroRNA Expression in Cancer. Int. J. Mol. Sci..

[9] Yang L., Cai N., Zhao L. (2020). MicroRNA-1 regulates the growth and chemosensitivity of breast cancer cells by targeting MEK/ERK pathway. J. BUON.

[10] Zhao Z., Sun W., Guo Z., Zhang J., Yu H., Liu B. (2020). Mechanisms of lncRNA/microRNA interactions in angiogenesis. Life Sci..

[11] Zhao L., Liu L., Dong Z., Xiong J. (2017). miR-149 suppresses human non-small cell lung cancer growth and metastasis by inhibiting the FOXM1/cyclin D1/MMP2 axis. Oncol. Rep..

[12] Ke Y., Zhao W., Xiong J., Cao R. (2013). miR-149 Inhibits Non-Small-Cell Lung Cancer Cells EMT by Targeting FOXM1. Biochem. Res. Int..

[13] He Y., Yu D., Zhu L., Zhong S., Zhao J., Tang J. (2018). miR-149 in Human Cancer: A Systemic Review. J. Cancer.

[14] Nasim F., Sabath B.F., Eapen G.A. (2019). Lung cancer. Med. Clin. N. Am..

[15] Latimer K.M., Mott T.F. (2015). Lung cancer: Diagnosis, treatment principles, and screening. Am. Fam. Physician.

[16] Rosell R., Dafni U., Felip E., Curioni-Fontecedro A., Gautschi O., Peters S., Massutí B., Palmero R., Aix S.P., Carcereny E. (2017). Erlotinib and bevacizumab in patients with advanced non-small-cell lung cancer and activating EGFR mutations (BELIEF): An international, multicentre, single-arm, phase 2 trial. Lancet Respir. Med..

[17] Chung C. (2016). Tyrosine kinase inhibitors for epidermal growth factor receptor gene mutation-positive non-small cell lung cancers: An update for recent advances in therapeutics. J. Oncol. Pharm. Pract..

[18] Hackler P.C., Reuss S., Konger R.L., Travers J.B., Sahu R.P. (2014). Systemic Platelet-activating Factor Receptor Activation Augments Experimental Lung Tumor Growth and Metastasis. Cancer Growth Metastasis.

[19] da Silva A., Chammas R., Lepique A.P., Jancar S. (2017). Platelet-activating factor (PAF) receptor as a promising target for cancer cell repopulation after radiotherapy. Oncogenesis.

[20] Sahu R.P., Ocana J.A., Harrison K.A., Ferracini M., Touloukian C.E., Al-Hassani M., Sun L., Loesch M., Murphy R.C., Althouse S.K. (2014). Chemotherapeutic agents subvert tumor immunity by generating agonists of platelet-activating factor. Cancer Res..

[21] Sahu R.P., Harrison K.A., Weyerbacher J., Murphy R.C., Konger R.L., Garrett J.E., Chin-Sinex H.J., Johnston M.E., Dynlacht J.R., Mendonca M. (2016). Radiation therapy generates platelet-activating factor agonists. Oncotarget.

[22] Zappa C., Mousa S.A. (2016). Non-small cell lung cancer: Current treatment and future advances. Transl. Lung Cancer Res..

[23] Pallis A.G., Gridelli C., Wedding U., Faivre-Finn C., Veronesi G., Jaklitsch M., Luciani A., O’Brien M. (2014). Management of elderly patients with NSCLC; updated expert’s opinion paper: EORTC Elderly Task Force, Lung Cancer Group and International Society for Geriatric Oncology. Ann. Oncol..

[24] Howe G.A., Xiao B., Zhao H., Al-Zahrani K.N., Hasim M.S., Villeneuve J., Sekhon H.S., Goss G.D., Sabourin L.A., Dimitroulakos J. (2016). Focal Adhesion Kinase Inhibitors in Combination with Erlotinib Demonstrate Enhanced Anti-Tumor Activity in Non-Small Cell Lung Cancer. PLoS ONE.

[25] Choi J., Kang M., Nam S.H., Lee G.-H., Kim H.-J., Ryu J., Cheong J.G., Jung J.W., Kim T.Y., Lee H.-Y. (2015). Bidirectional signaling between TM4SF5 and IGF1R promotes resistance to EGFR kinase inhibitors. Lung Cancer.

[26] Lin C., Qin Y., Zhang H., Gao M.Y., Wang Y.F. (2018). EGF upregulates RFPL3 and hTERT via the MEK signaling pathway in non small cell lung cancer cells. Oncol. Rep..

[27] Chen J., Lan T., Zhang W., Dong L., Kang N., Zhang S., Fu M., Liu B., Liu K., Zhang C. (2015). Platelet-activating factor receptor-mediated PI3K/AKT activation contributes to the malignant development of esophageal squamous cell carcinoma. Oncogene.

[28] Lordan R., Tsoupras A., Zabetakis J. (2019). The Potential Role of Dietary Platelet-Activating Factor Inhibitors in Cancer Prevention and Treatment. Adv. Nutr..

[29] Chen J., Lan T., Zhang W., Dong L., Kang N., Zhang S., Fu M., Liu B., Liu K., Zhan Q. (2015). Feed-Forward Reciprocal Activation of PAFR and STAT3 Regulates Epithelial-Mesenchymal Transition in Non-Small Cell Lung Cancer. Cancer Res..

[30] Cruz C.S.D., Tanoue L.T., Matthay R.A. (2011). Lung cancer: Epidemiology, etiology, and prevention. Clin. Chest Med..

[31] Chauhan S.J., Thyagarajan A., Chen Y., Travers J.B., Sahu R.P. (2020). Platelet-Activating Factor-Receptor Signaling Mediates Targeted Therapies-Induced Microvesicle Particles Release in Lung Cancer Cells. Int. J. Mol. Sci..

[32] Raychaudhuri P., Park H.J. (2011). FoxM1: A master regulator of tumor metastasis. Cancer Res..

[33] Katoh M., Igarashi M., Fukuda H., Nakagama H., Katoh M. (2013). Cancer genetics and genomics of human FOX family genes. Cancer Lett..

[34] Kim I.M., Ackerson T., Ramakrishna S., Tretiakova M., Wang I.C., Kalin T.V., Major M.L., Gusarova G.A., Yoder H.M., Costa R.H. (2006). The Forkhead Box m1 transcription factor stimulates the proliferation of tumor cells during development of lung cancer. Cancer Res..

[35] Kong X., Li L., Li Z., Le X., Huang C., Jia Z., Cui J., Huang S., Wang L., Xie K. (2013). Dysregulated expression of FOXM1 isoforms drives progression of pancreatic cancer. Cancer Res..

[36] Huang C., Xie D., Cui J., Li Q., Gao Y., Xie K. (2014). FOXM1c promotes pancreatic cancer epithelial-to-mesenchymal transition and metastasis via upregulation of expression of the urokinase plasminogen activator system. Clin. Cancer Res..

[37] Huang C., Zhang X., Jiang L., Zhang L., Xiang M., Ren H. (2019). FoxM1 Induced Paclitaxel Resistance via Activation of the FoxM1/PHB1/RAF-MEK-ERK Pathway and Enhancement of the ABCA2 Transporter. Mol. Ther. Oncolytics.

[38] Pei Y., Barber L.A., Murphy R.C., Johnson C.A., Kelley S.W., Dy L.C., Fertel R.H., Nguyen T.M., Williams D.A., Travers J.B. (1998). Activation of the epidermal platelet-activating factor receptor results in cytokine and cyclooxygenase-2 biosynthesis. J. Immunol..

[39] Sahu R.P. (2015). Expression of the platelet-activating factor receptor enhances benzyl isothiocyanate-induced apoptosis in murine and human melanoma cells. Mol. Med. Rep..

[40] Crystal Violet Staining. https://openwetware.org/wiki/Crystal_Violet_Staining.

