# Supplementary material for: Effects of miRNA-149-5p and Platelet-Activating Factor-Receptor Signaling on the Growth and Targeted Therapy Response on Lung Cancer Cells
Source: Int J Mol Sci. 2022 Jun 17;23(12):6772. doi: 10.3390/ijms23126772 (PMC9223644; doi:10.3390/ijms23126772)
Supplement: Supplementary file 1 [file ijms-23-06772-s001.zip › ijms-1737937-supplementary.pptx]

## Slide 1
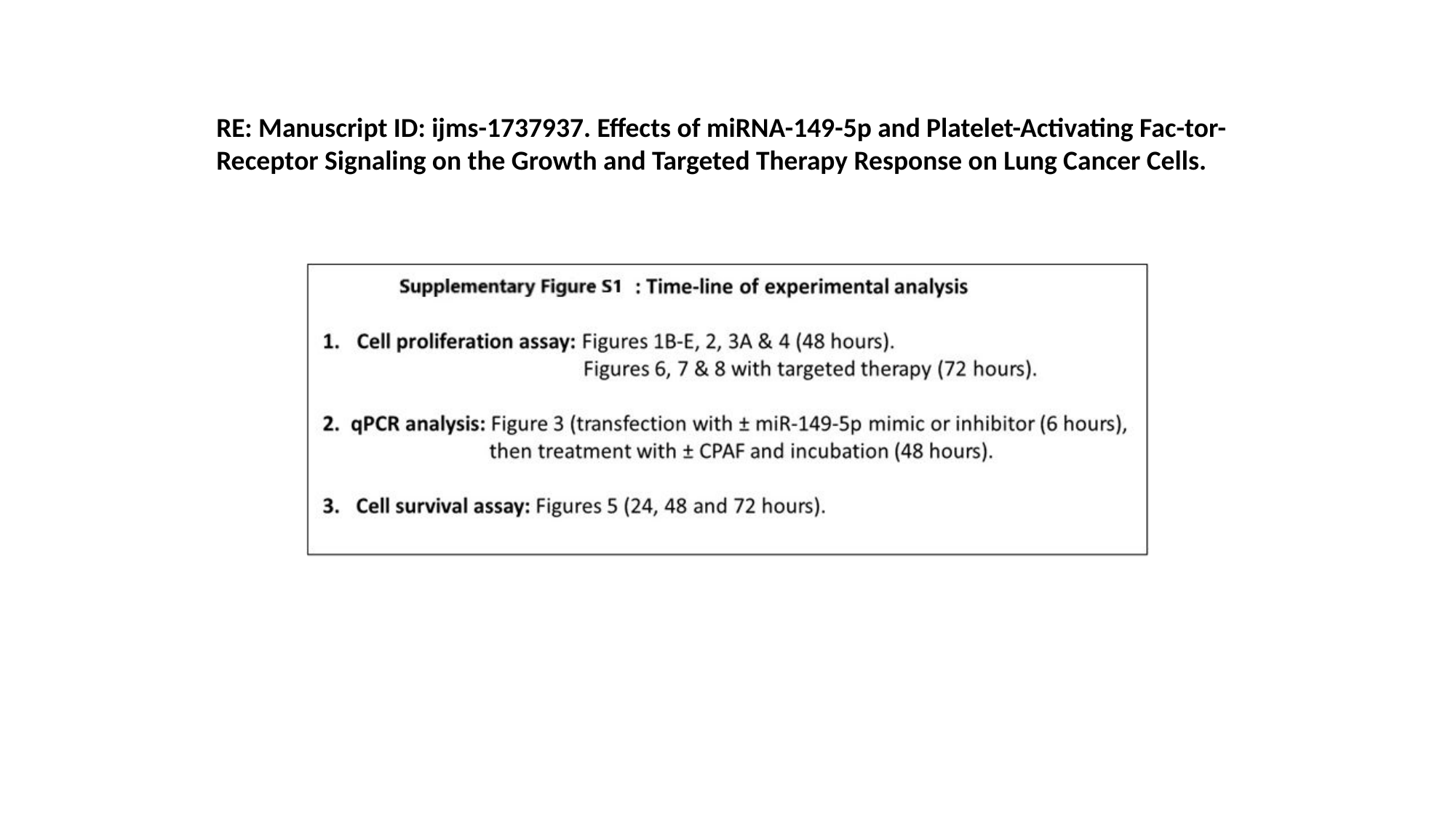

RE: Manuscript ID: ijms-1737937. Effects of miRNA-149-5p and Platelet-Activating Fac-tor-Receptor Signaling on the Growth and Targeted Therapy Response on Lung Cancer Cells.
